# Infantile orbital abscess caused by community-acquired methicillin-resistant *Staphylococcus aureus*

**DOI:** 10.1007/s12348-011-0030-1

**Published:** 2011-07-26

**Authors:** Wen-Chia Chung, Hung-Jung Lin, Ning-Ping Foo, Kuo-Tai Chen

**Affiliations:** 1 Emergency Department, Chi-Mei Medical Center, 901 Chung-Hwa Road, Yung Kang, Tainan, 710 Taiwan; 2 Department of Biotechnology, Southern Tainan University of Technology, Tainan, Taiwan; 3 Department of Emergency Medicine, Chi-Mei Medical Center, Liouying, Tainan, Taiwan; 4 Department of Emergency Medicine, Taipei Medical University, Taipei, Taiwan

**Keywords:** Orbital abscess, Infant, Methicillin-resistant *Staphylococcus aureus*, Computed tomography, Surgery

## Abstract

An orbital abscess is a severe infectious disease of the eye and carries the risks of visual loss and intracranial complications. Timely treatment in an infant is crucial in order to save sight and can prevent unnecessary morbidity and mortality. We report a case of an orbital abscess in an infant who underwent surgical drainage and medical management. The unique clinical and radiological features are presented in this report. In addition, methicillin-resistant *Staphylococcus aureus* was isolated from the pus culture. The case reminds us that, before obtaining the result of the pus culture, with the emergence of community-acquired methicillin-resistant *S. aureus*, physicians should consider empirical antibiotic therapy, which is effective against methicillin-resistant *S. aureus*.

## Introduction

Acute ethmoid and maxillary sinusitis are the most common causes of an orbital abscess [6, 8]. *Streptococcus* species, *Haemophilus influenzae*, and *Staphylococcus aureus* are organisms frequently isolated from an abscess [1, 3, 4]. We report a case of an infant who was admitted for left orbital abscess who underwent surgical drainage of the abscess. The culture of the pus revealed methicillin-resistant *S. aureus* (MRSA), which highlights the increasing prevalence of community-acquired MRSA infections.

## Case report

**Fig. 1:**
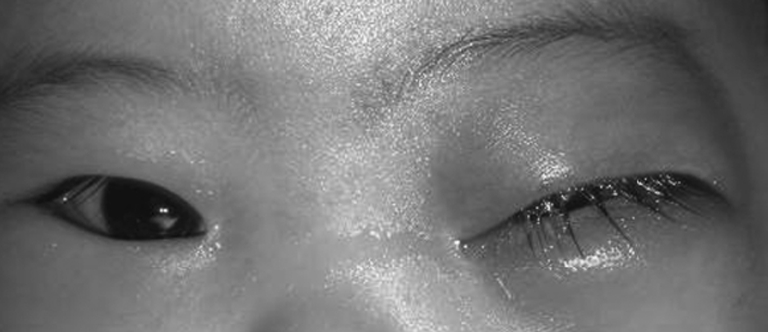
The patient's left eye was swollen with increased tears

**Fig. 2:**
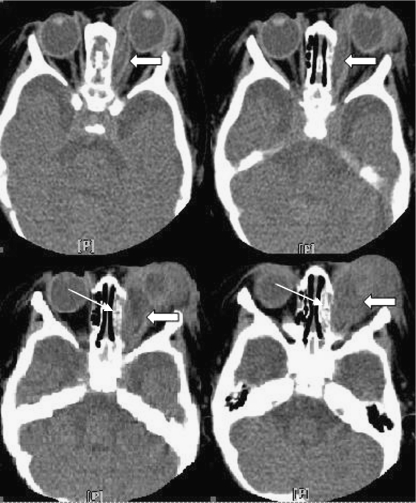
CT images demonstrated left ethmoid sinusitis (*thin white arrow*) and a left orbital abscess with adjacent soft tissue swelling (*thick white arrow*)

A previously healthy 5-month-old female infant was brought to the emergency department for progressive proptosis. Her parents denied any history of trauma or fever episode in the previous 1 month, and there was no regular medication being taken. She was a full-term infant without any birth defects, and vaccinations were received regularly. Physical examination revealed a well-developed infant with a weight of 3,200 g, a pulse of 100 beats per minute, a respiratory rate of 24 per minute, and a temperature of 36°C. Her left periorbital region showed swelling, redness, and tenderness to palpation. In addition, the conjunctiva of the left eye was swollen, and increased tears were observed (Fig. 1). Laboratory investigations revealed a white blood cell count of 36,100/μL and a C-reactive protein of 42.5 mg/dL. Soft tissue infection of the left periorbital region was suspected, and therefore, the patient underwent an orbital computed tomography (CT) scan. CT images demonstrated a heterogenous lesion in the left orbit with adjacent soft tissue swelling (Fig. 2) and opacified left ethmoid and maxillary sinuses, which was diagnosed as an orbital abscess and sinusitis of the ethmoid and maxillary sinuses.

The patient underwent decompression surgery of the left orbit using an external approach, and 10 ml of pus was drained out. Bacteriological culture of the pus revealed MRSA, and the results from blood culture were negative. After a 10-day course of intravenous vancomycin treatment, the patient was discharged home without permanent visual damage upon follow-up.

## Discussion

Infections of the orbital and periorbital regions usually present as periorbital swelling and, in most cases, result from complications of acute sinusitis. However, trauma, insect bites, conjunctivitis, dacryocystitis, and blepharitis are alternative causes [1–3]. *Staphylococcus*, *Streptococcus*, and *H. influenzae* account for the majority of infected pathogens [1, 3, 4]. Currently, community-acquired MRSA has become increasingly prevalent. McKinley et al. found that MRSA represented 73% of all pediatric *S. aureus* orbital infections [5]. Empirical intravenous antibiotic treatment should have coverage against *H. influenzae*, streptococcal and staphylococcal species. A CT scan is the most useful tool to evaluate the extent of the infection and the location of the accumulated pus [4, 6]. In addition, a CT scan can assess the sinuses and explore the other causative factors.

Controversy exists about the medical or surgical management of orbital abscesses in pediatric patients. Some otolaryngologists advocate medical treatment initially, and surgery is reserved for nonresponders, whereas others suggest immediate surgical drainage whenever the CT scan shows the presence of an orbital abscess. The objectives in the surgery of orbital abscess are to release the pressure on the orbit, drain the abscess, and obtain a specimen for culture [4, 6, 8]. Because successful medical treatment relies on normal visual examination, and inadequate treatment might lead to the loss of vision [4, 6, 8], early surgical intervention is indicated in infantile patients due to the inability to perform a reliable and serial ophthalmologic examination.

The majority of the results from blood cultures are negative in patients with orbital abscess [7]. Therefore, the choice of antibiotics relies on the specimen of pus culture. In our case, the organism of the pus culture was MRSA, which reminds physicians of the increasing prevalence of community-acquired MRSA infection [1, 2, 5]. If a patient's response to empirical antibiotics is inappropriate, and surgical drainage is impeded, we suggest adding antibiotics which are effective against MRSA in order to extend the coverage of medical treatment. Trimethoprim or clindamycin plus a second- or third-generation cephalosporin is a reasonable initial regimen for the coverage of MRSA and pathogens associated with rhinosinusitis [3, 5].

Physicians need to be aware of the clinical presentations and classical CT findings of orbital abscesses. Early surgical drainage of an orbital abscess might be indicated in infantile patients to avoid the complications of visual loss. Due to the emergence of community-acquired MRSA, before obtaining the results from a pus culture, physicians should consider empirical antibiotics that are effective against MRSA.

## References

[1] Asbell PA, Colby KA, Deng S (2008). Ocular TRUST: nationwide antimicrobial susceptibility patterns in ocular isolates. Am J Ophthalmol.

[2] Bertino JS (2009). Impact of antibiotics resistance in the management of ocular infections: the role of current and future antibiotics. Clin Ophthalmol.

[3] Hauser A, Fogarasi S (2010). Periorbital and orbital cellulitis. Pediatr Rev.

[4] Howe L, Jones NS (2004). Guidelines for the management of periorbital cellulitis/abscess. Clin Otolaryngol Allied Sci.

[5] McKinley SH, Yen MT, Miller AM, Yen KG (2007). Microbiology of pediatric orbital cellulitis. Am J Ophthalmol.

[6] Rahbar R, Robson CD, Petersen RA (2001). Management of orbital subperiosteal abscess in children. Arch Otolaryngol Head Neck Surg.

[7] Schramm VL, Curtin HD, Kennerdell JS (1982). Evaluation of orbital cellulitis and results of treatment. Laryngoscope.

[8] Tanna N, Preciado DA, Clary MS, Choi SS (2008). Surgical treatment of subperiosteal orbital abscess. Arch Otolaryngol Head Neck Surg.

